# Supplementary material for: Online Extraction Followed by LC–MS/MS Analysis of Lipids in Natural Samples: A Proof-of-Concept Profiling Lecithin in Seeds
Source: Foods. 2023 Jan 7;12(2):281. doi: 10.3390/foods12020281 (PMC9858076; doi:10.3390/foods12020281)
Supplement: Supplementary file 1 [file foods-12-00281-s001.zip › foods-2070148-supplementary.pdf]

Supplementary material

Online Extraction Followed by LC-MS/MS Analysis of Lipids in Natural Samples: A Proof-of-Concept Profiling Lecithin in Seeds

João V. B. Borsatto, Edvaldo V. S. Maciel, Alejandro Cifuentes, Fernando M. Lanças

1. SiGO-C18ec columns construction

The SIGO-C18ec LC columns hardware is composed by a 100 mm length fused silica capillary internally covered by a stainless-steel tube (glass lined stainless-steel tube) of 1/16 inches o.d. and 0.5 mm i.d.

1-2 mm glass wool filter disc of 1/16 inches of diameter and 0.2 µm of porosity were used as the frits. The frit was assembled to the LC columns by a set of Valco unions, nuts, and ferrules for 1/16 inches tubing.

15 mg of SiGO-C18ec particles were previously washed with 1 ml of acetone to remove non-bounded graphene sheets. After the decantation, the supernatant was collected and discarded to eliminate non bonded graphene sheets. The SiGO-C18ec particles were dried at room temperature for 24h.

The column was packed by a slurry-packing process performed at a hydro-pneumatic pump DSHF-300 (Haskel, Burbank, USA) at 9000 kPa (900 bar) for 1 hour, followed by 0.5 hour of pressure re-equilibration. The packing solvent was ultra-pure water, while the SiGO-C18ec was suspended in a mixture of IPA: THF 4:1 before inserting into the packing chamber.

2. Scheme of the OLE-LC-MS set up

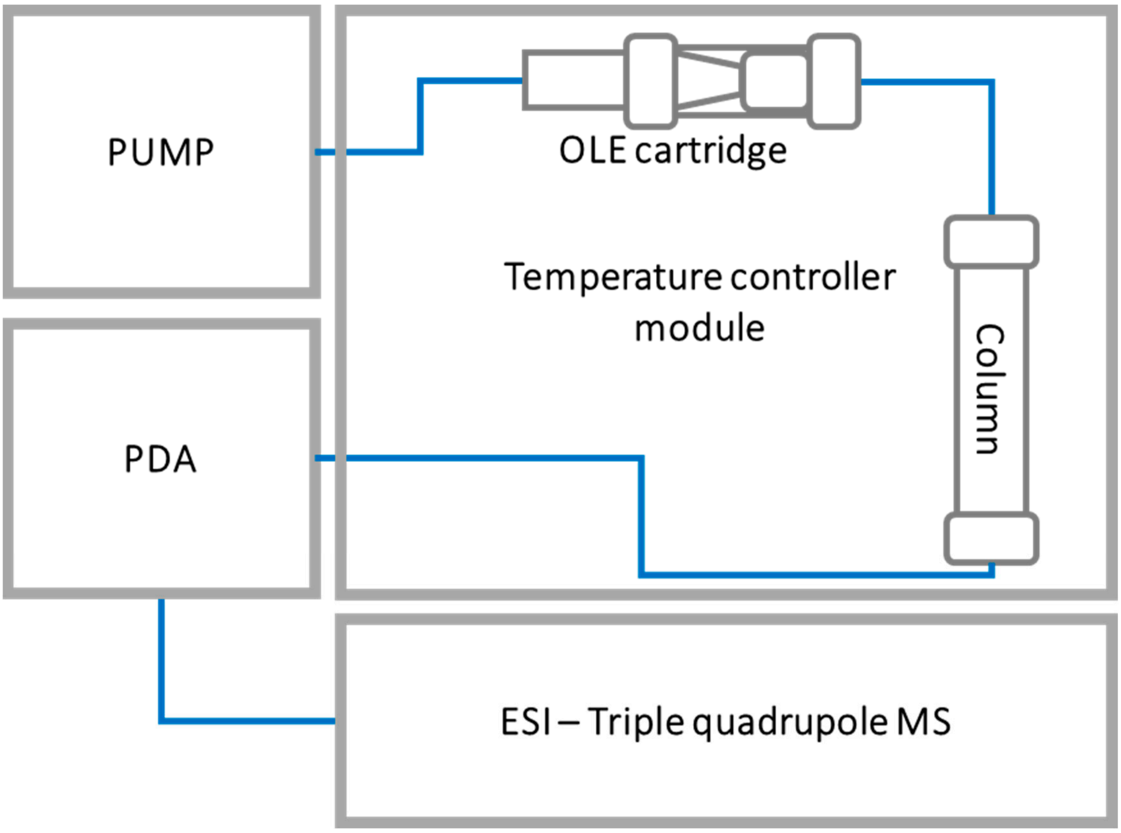

Figure S1. Set up of the OLE-LC-MS system.

3. MS spectra of each peak of lecithin before 20 min.

Table S1. Retention time and collected ions for each peak observed in the lecithin standard (including only m/z signals higher than 10% of the base peak).

tR	*m/z 1*	*m/z 2*	*m/z 3*	*m/z 4*	*m/z 5*	*m/z 6*	*m/z 7*	*m/z 8*	*m/z 9*	*m/z 10*
8.4	184	337	502	520	1039					
9.9	184	506	524	782	814	830				
10.4	184	780	796	812						
10.8	184	597	613	756	780	796				
13.6	184	575	601	758	782					
15.2	184	306	575	575	599	758	782	842		
16	184	313	575	599	758	782				

4. Retention time and MS spectra from the OLE-LC-MS blank .

Table S2. Retention time and collected ions for each peak observed in a blank sample

(only m/z signals higher than 10% of the base peak).

tR	*m/z 1*	*m/z 2*	*m/z 3*	*m/z 4*	*m/z 5*	*m/z 6*	*m/z 7*	*m/z 8*	*m/z 9*	*m/z 10*
6.8	272	565								
8.2	303	321	338	907						
10.4	313	551	607	663	685	907				
10.9	313	467	537	551	575	589	607	663	749	907
12.5	313	467	537	551	575	589	607	663	749	907
14.5	313	467	523	551	577	603	663	881	907	
15.7	313	495	551	607	663	685	823			
23.5	495	551	607	663	832					
